# Analysed cap mesenchyme track data from live imaging of mouse kidney development

**DOI:** 10.1016/j.dib.2016.08.053

**Published:** 2016-09-01

**Authors:** James G. Lefevre, Alexander N. Combes, Melissa H. Little, Nicholas A. Hamilton

**Affiliations:** a Division of Genomics of Development and Disease, Institute for Molecular Biosciences, The University of Queensland, Brisbane, QLD 4072, Australia; b Department of Anatomy & Neuroscience, University of Melbourne, Melbourne, 3010 VIC, Australia; c Murdoch Childrens Research Institute, Flemington Rd, Parkville, Melbourne, 3052 VIC, Australia; d Department of Paediatrics, The University of Melbourne, Melbourne, 3010 VIC, Australia; e Division of Cell Biology and Molecular Medicine, Institute for Molecular Bioscience, The University of Queensland, Brisbane, QLD 4072, Australia

**Keywords:** Kidney development, Cap mesenchyme, Cell migration, Image analysis, Mathematical modelling

## Abstract

This article provides detailed information on manually tracked cap mesenchyme cells from timelapse imaging of multiple *ex vivo* embryonic mouse kidneys. Cells were imaged for up to 18 h at 15 or 20 min intervals, and multiple cell divisions were tracked. Positional data is supplemented with a range of information including the relative location of the closest ureteric tip and a correction for drift due to bulk movement and tip growth. A subset of tracks were annotated to indicate the presence of processes attached to the ureteric epithelium. The calculations used for drift correction are described, as are the main methods used in the analysis of this data for the purpose of describing cap cell motility. The outcomes of this analysis are discussed in “Cap mesenchyme cell swarming during kidney development is influenced by attraction, repulsion, and adhesion to the ureteric tip” (A.N. Combes, J.G. Lefevre, S. Wilson, N.A. Hamilton, M.H. Little, 2016) [1].

**Specifications Table**

**Table:** 

Subject area	*Biology*
More specific subject area	*Cell motility in kidney development*
Type of data	*Tables*
How data was acquired	*Confocal Microscope, Zeiss 710 and 780, analysis in Imaris (Bitplane 8.0.1) and R*
Data format	*Analysed*
Experimental factors	*Cellular compartments within the developing mouse kidney were labeled with transgenic reporters and imaged in organ culture using confocal microscopy*
Experimental features	*Quantitative analysis of cell migration data, computed information*
Data source location	*Institute for Molecular Biosciences, The University of Queensland, Brisbane, QLD, Australia*
	*Murdoch Childrens Research Institute, Melbourne, VIC, Australia*
Data accessibility	*Data is included in this article*

**Value of the data**

• Timelapse imaging dataset from complex developing organ including methods to correct for drift and compute relationships between cells and local features in a dynamic environment.
• Includes data on position and movement of cap mesenchyme cells relative to ureteric tip.
• Available for further analysis and modelling of cell motility.
• Provides benchmark for studying mutant phenotypes.

## 1 Data

The data consists of 3 tables formatted as Excel files, and an additional Excel file containing detailed metadata. Supplementary Table 1 gives the primary cap mesenchyme cell dataset summarised in Table 1, Supplementary Table 2 gives the same dataset with position and derived fields transformed according to the drift correction described below, while Supplementary Table 3 contains tip extremity tracks that were used for drift correction. Each table row corresponds to a single measurement and contains sample, crop, track and track branch identifiers as well as the time step, position and additional calculated data fields.

## 2 Experimental design, materials and methods

See [1] for information on mouse strains, selective labelling, and imaging. Kidneys were cultured as previously described [2] and imaged ~24 h after induction.

Image analysis was performed in Imaris (Bitplane). In some cases the image file for a sample was partitioned into multiple crops prior to analysis. Cap mesenchyme cells and tip ends were manually identified and tracked using the ‘Spots’ feature. Branching was permitted in cell tracks to represent mitosis, but not in tip tracks. Time step, 3D positional information, and track identifiers were exported into csv files. A representation of the tip surface and volume was also produced in Imaris using automatic volume rendering for tip expressed *Hoxb7-EGFP*. The volume contained within this surface was then filled with arbitrary points using the ‘Spots’ feature, so that the spot positions fully defined the tip surface and volume of the ureteric around which the migrating cells were being tracked. These spot positions were exported without track information. Relevant data fields were loaded into R, annotated, and collated across samples.

## 3 Identification of branch points and unbranched track sections

In the case of tracks that branched due to cell mitosis, the track identifier provided by Imaris (version 8.0.1) did not distinguish between the daughter cells, and branch points were not identified. To remedy this deficit, for each branched track the cell positions at consecutive time points were associated such that (1) each cell position was matched with at most 1 position at the previous time step and 2 positions at the following time step, (2) the number of track bifurcations and terminations was minimised and, (3) subject to 1 and 2, the total distance between matched positions was minimised. The resulting track branches were plotted and visually verified. Branch points were identified and branched tracks were separated at these points for analysis.

## 4 Identification of position relative to tip

For each tracked cell at each time step, the position (prior to drift correction) was compared to the set of spot positions representing the tip volume at the given time step. The closest spot was identified and the vector describing the position of the spot relative to the cell was recorded. A similar process was used to identify the nearest tip end track (the manually tracked extremities of the ureteric tips, used in drift correction), recording distance and tip track id, but this was not found to be of predictive value in motility analysis.

## 5 Drift correction

Imaged samples typically contained multiple ureteric tips growing in various directions. Cap cells were often associated with a single tip with which they appeared to track, but there were also many cells that occupied intermediate positions or moved between tips. The aim of drift correction was to subtract the effect of movement with the tips and also any bulk movement of the sample. A custom protocol was developed to adjust for this spatially heterogeneous tissue movement. For each interval between consecutive time points, the movement of each CM cell and each tip was calculated. For each cell, the tracked tip positions were assigned weights proportional to the inverse square distance from the cell to the tip at the given time step. The weighted average of the tip movements was then subtracted from the cell movement to give the drift corrected cell movement in the given interval. When the niche of a CM cell was unambiguous, this algorithm assigned predominant weight to the associated tip track, while providing smooth transitions when the niche was ambiguous or changed over time. Any bulk movement of the sample that perturbed all tips was carried through to the weighted average and subtracted in the drift correction. Drift correction was applied to horizontal movement only. Drift corrected positions were calculated by retaining the original position for each track at the first time step, and deriving subsequent positions by adding drift corrected movements.

## 6 Attachment to ureteric epithelium

Attachment of cells to the tip surface, indicated by elongated morphology, was manually identified in Imaris for a subset of tracks. Periods of attachment were identified by a track id and first and last time step. Tracks without observed attachment were also specified by track id. This information was mapped onto the data in R, allowing individual observations (spots) to be annotated as attached or free. Due to the limited z resolution attachment may be obscured when the cell is located directly above or below the nearest tip surface. To account for this possibility, the relative position of the nearest tip spot was used. If the vertical angle of this direction vector was less than 45° the spot was considered to be horizontally located relative to the tip, and the proportion of attached cells was assessed using this subset of the data. All tracks in samples 13 and 14 were annotated for attachment where imaging was sufficiently clear; 116 of 169 unbranched tracks were annotated, containing 2922 of the 4179 spots for these two samples. This represents 11.5% of the complete data. There were 25 periods of attachment identified over 23 unbranched tracks. Of the 2922 annotated spots, 1975 were located horizontally relative to the nearest tip.

## 7 Mean squared displacement and autocorrelation

Directionality of movement was assessed using mean squared displacement (MSD) and velocity autocorrelation. Mean squared displacement (MSD) is the average squared displacement of a cell over a given time difference, where displacement is the straight line distance from the initial position rather than the entire length of the path travelled. A linear relationship between time difference and MSD indicates Brownian motion [3], with the slope giving the diffusion rate. An upwards curving plot indicates persistent directed movement, while a downwards curve indicates a confinement effect. Comparing MSD between raw and drift corrected data allowed us to quantify the extent to which persistent directed movement was associated with tip growth. Velocity autocorrelation was used to measure short term persistence of movement direction.

For a given unbranched track with position p(t) at time step *t*, and a given time difference τ, the mean squared displacement is

$$\bar{{\left|p\left(\tau +t\right)-p\left(t\right)\right|}^{2}}$$

and the autocorrelation is

$$\bar{\left(p\left(t+1\right)-p\left(t\right)\right)∙\left(p\left(\tau +t+1\right)-p\left(\tau +t\right)\right)}/\bar{{\left|p\left(t+1\right)-p\left(t\right)\right|}^{2}},$$

where averages are taken over all values of *t* for which data is available. For each time difference τ, MSD and autocorrelation were then averaged across all cell tracks. Since this aggregation is done for a specific time difference, it was necessarily performed separately for the data with 15 and 20 min time steps. The 20 min data is shown in Fig. 2 (Combes et al. [1] in press), while the 15 min data was used to test consistency, showing a very similar pattern (data not shown).

Resolution in the *z* (vertical) axis was significantly poorer than in the horizontal plane. This caused a “jitter” effect, in which negative velocity autocorrelation was seen between consecutive time steps, in the *z* direction only. Single time step movement was also greater in the *z* direction, with spikes in the distribution. Since this was clearly an image analysis artefact, velocity autocorrelation and the instantaneous speed distribution were calculated in the horizontal plane only. Other calculations such as MSD are insensitive to this short term noise, and are calculated in 3D.

## 8 Cell speed heterogeneity

To assess heterogeneity in speed between tracks, a linear regression was performed including sample id as a possible confounder, using the entire data set detailed above. The instantaneous horizontal speed was regressed firstly against the sample and then against both sample and track (categorical variables). The ANOVA function in R was used to compare these 2 models, indicating that the model including track should be preferred (*p*<2.2e−16). This was interpreted as strong evidence for track heterogeneity in speed. The residual sum of squares was reduced by 8.9% in the second model compared to the first, which was interpreted as the percentage of speed variability accounted for by this heterogeneity.

## 9 Tip attraction and repulsion

To assess attraction or repulsion from the tip surface, an unbiased test was performed by considering each spot (a single cell at a single time) and comparing two vectors: the relative position of the nearest tip spot at that time and the (drift corrected) movement of the cell over the subsequent time step. The component of net movement in the direction defined by the nearest tip spot was considered to be the movement towards or away from the tip surface. This metric was used in preference to the change in tip distance due to the complex tip geometry; random motion will not necessarily result in an equal chance of increase or decrease in the distance to the tip. Cell positions within 1 µm of the edge of the image section were excluded from this analysis as their possible subsequent movement is constrained, leading to possible bias (outwards movement may cause them to move out of the image area). Although individual cell movements may be greater than 1 µm, the direction of median movement will be robust against the truncation of larger individual movements. For cells initially more than 10 µm from the nearest tip point, while mean overall movement in any direction was 4.1 µm, net directional movement was towards the time by 0.17 µm per time step (median 0.07 µm; *p*=9.1e−14, *t*-test). Conversely, for cells within 10 µm of the nearest tip point, overall movement was 3.6 um in any direction by net movement was away from the tip by 0.24 µm (median 0.17 µm; *p*=3.4e−16, *t*-test). These results were each confirmed with a single sample, two sided *t*-test, giving *p*=3.36e−16 (*n*=6865) for tip distances <10 µm and *p*=9.078e−14 (*n*= 14,080) for distance >10 µm.

After transitioning from repulsion at the tip surface to attraction beyond a distance of 10 µm, the level of attraction appears to be approximately constant beyond about 15 µm. This was confirmed by a linear regression of movement towards tip against initial tip distance using all cases in which the initial tip distance exceeded 15 µm, finding no evidence of a relationship (*p*=0.99).

## 10 Tip distance modelling

A steady state convection-diffusion model was fitted to the observed distribution of tip distances, in order to test whether this pattern of attraction and repulsion was sufficient to maintain the cap cells in proximity to the ureteric tip. We assume that the movement of cap cells towards or way from the tip surface is the combination of an undirected diffusion D (independent of tip distance), and a convection or directed velocity v(d), which is a function of tip distance d (positive values correspond to repulsion, negative values to attraction to the tip).

The distribution of cap cells with distance and time, y(d,t), satisfies

$$D\frac{\partial^{2}y}{\partial x^{2}}=v\frac{\partial y}{\partial x}.$$

Although individual cells move significantly, we assume that the overall distribution of tip distances is approximately stable, and consider the well-known steady state solution

$$y\left(d\right)=y\left(0\right)e^{\int_{0}^{d}k\left(u\right)\mathrm{du}},$$

where $k\left(d\right)=\frac{v\left(d\right)}{D}.$ The function k(d) was assumed to have a form with a constant value of $k_{1}$ for $d<d_{1}$, a constant value of $k_{2}$ for $d>d_{2}$, and vary linearly between $d_{1}$ and $d_{2}$. This four parameter model was fitted to the set of observed tip distances using maximum likelihood. The fitted model gave $d_{1}=0.005\pm 0.381$, $d_{2}=14.95\pm 0.18$, $k_{1}=0.293\pm 0.010$, $k_{2}=-0.098\pm 0.001$. Since $d_{1}$ was not significantly different from 0 we used the simplified model

$$k\left(d\right)=\{\begin{array}{ll} 0.293-0.026d, & 0\leq d\leq 14.95,\\ -0.098, & d>14.95. \end{array}$$

## Figures and Tables

**Table 1:** Data Summary.

*Sample id*	*Crops*	*Time step*	*Experiment duration*	*Tracks*	*Analysis tracks*	*Total observations*
		*(minutes)*	*(hours)*	*(may be branched)*	*(unbranched)*	*(spots)*
1	1	15	11.75	1	3	95
4	1	15	12.00	102	118	3753
5	1	15	17.25	5	9	474
6	1	15	18.00	10	14	707
11	5	15	16.25	249	281	8593
13	1	20	18.67	38	92	2262
14	1	20	18.00	47	77	1917
16	4	20	18.00	125	189	5295
17	4	20	17.67	47	69	2391
**total**	**19**			**624**	**852**	**25,487**

## Footnotes

Appendix A Supplementary data associated with this article can be found in the online version at doi:10.1016/j.dib.2016.08.053.
